# Evaluating the Bone Tissue Regeneration Capability of the Chinese Herbal Decoction *Danggui Buxue Tang* from a Molecular Biology Perspective

**DOI:** 10.1155/2014/853234

**Published:** 2014-09-11

**Authors:** Wen-Ling Wang, Shi-Yuan Sheu, Yueh-Sheng Chen, Shung-Te Kao, Yuan-Tsung Fu, Tzong-Fu Kuo, Kuo-Yu Chen, Chun-Hsu Yao

**Affiliations:** ^1^School of Chinese Medicine, China Medical University, Taichung 40402, Taiwan; ^2^Department of Chinese Internal Medicine, China Medical University Hospital, Taichung 40402, Taiwan; ^3^School of Medicine, Chung Shan Medical University, Taichung 40201, Taiwan; ^4^Department of Integrated Chinese and Western Medicine, Chung Shan Medical University Hospital, Taichung 40201, Taiwan; ^5^Department of Biomedical Imaging and Radiological Science, China Medical University, Taichung 40202, Taiwan; ^6^Department of Chinese Medicine, Taichung Tzu Chi Hospital, The Buddhist Tzu Chi Medical Foundation, Taichung 40427, Taiwan; ^7^Department of Veterinary Medicine, School of Veterinary Medicine, National Taiwan University, Taipei 10617, Taiwan; ^8^Department of Chemical and Materials Engineering, National Yunlin University of Science and Technology, Yunlin 64002, Taiwan; ^9^Department of Biomedical Informatics, Asia University, Taichung 41354, Taiwan

## Abstract

Large bone defects are a considerable challenge to reconstructive surgeons. Numerous traditional Chinese herbal medicines have been used to repair and regenerate bone tissue. This study investigated the bone regeneration potential of *Danggui Buxue Tang* (*DBT*), a Chinese herbal decoction prepared from *Radix Astragali* (*RA*) and *Radix Angelicae Sinensis* (*RAS*), from a molecular biology perspective. The optimal ratio of *RA* and *RAS* used in *DBT* for osteoblast culture was obtained by colorimetric and alkaline phosphatase (ALP) activity assays. Moreover, the optimal concentration of *DBT* for bone cell culture was also determined by colorimetric, ALP activity, nodule formation, Western blotting, wound-healing, and tartrate-resistant acid phosphatase activity assays. Consequently, the most appropriate weight ratio of *RA* to *RAS* for the proliferation and differentiation of osteoblasts was 5 : 1. Moreover, the most effective concentration of *DBT* was 1,000 *μ*g/mL, which significantly increased the number of osteoblasts, intracellular ALP levels, and nodule numbers, while inhibiting osteoclast activity. Additionally, 1,000 *μ*g/mL of *DBT* was able to stimulate p-ERK and p-JNK signal pathway. Therefore, *DBT* is highly promising for use in accelerating fracture healing in the middle or late healing periods.

## 1. Introduction

Bone injuries are commonly caused by trauma, infection, diseases, or tumor removal. Clinically, bone begins to repair itself within weeks following injury and lasts for months. The healing process includes three stages: inflammation, repair, and remodeling. Bone remodeling is dynamically equilibrated by bone-forming osteoblasts and bone-resorbing osteoclasts for several months up to 1 year. Bone mineralization generally allows more time to proceed with healing in order to comply with changing skeletal growth for mechanical requirements. Many clinical and animal studies have demonstrated that traditional Chinese medicines have beneficial therapeutic effects on bone fracture healing [1–4]. Therefore, the biochemical effects of traditional Chinese medicines using an* in vitro* bone cell culture model have received considerable attention [5–7].

*Danggui Buxue Tang* (*DBT*), a Chinese herbal decoction consisting of Huangqi (*Radix Astragali*,* RA*) and Danggui (*Radix Angelicae Sinensis*,* RAS*) with a weight ratio of 5 : 1, is widely used for menopausal women to nourish qi and blood. According to recent pharmacological studies,* DBT* can enhance cardiovascular circulation, prevent osteoporosis, increase antioxidant activity, and stimulate and regulate immune functions [8, 9]. Additionally,* RA* and* RAS* can promote the proliferation of bone cells, induce bone formation, inhibit bone resorption in patients [10], and increase the proliferation and differentiation of the osteoblasts [11, 12].

This study examined the biological effects of different ratios of* RA* to* RAS* in* DBT* and various* DBT* concentrations on bone cell activities via* in vitro* cell culture. The possible pharmacological mechanism of the* DBT* to facilitate bone regeneration was also investigated.

## 2. Materials and Methods

### 2.1. Plant Materials and DBT Preparation

Fresh roots,* RA* (*A. membranaceus* var.* mongholicus*) and* RAS* (*A. sinensis*), were purchased from Chuang Song Zong Pharmaceutical Co. (Kaohsiung, Taiwan). Their identity was confirmed by experts in pharmacognosy.* DBT* was prepared using a method described previously [13]. The extraction process of the crude drugs was performed under strict quality control. Briefly,* RA* and* RAS* were boiled separately in 6 volumes of water for 1 h. The residue from first extraction was then boiled in 8 volumes of water for 1.5 h. The aqueous extracts were combined, filtered to remove insoluble debris, and stored at −20°C. The biological activities of* DBT* extracts were evaluated by preparing* RA* and* RAS* at ratios of 1 : 5, 2 : 1, 5 : 1, and 10 : 1. Finally, various concentrations of* DBT* were prepared and stored at 4°C until the* in vitro* assays. The culture medium without* DBT* was used as a control.

### 2.2. Cell Culture

The human osteoblast-like cell line MG-63 (BCRC number 60279) was obtained from the Food Industry Research and Development Institute (FIRDI, Hsinchu, Taiwan). Cells were grown in Modified Eagle's medium (MEM, Gibco-BRL, Rockville, MD, USA) supplemented with 10% fetal bovine serum (FBS, Gibco, Grand Island, NY, USA) and 1% penicillin/streptomycin (Gibco) in a humidified 5% CO_2_ incubator at 37°C. Cells were tested after growth to 80% confluence. Cultured MG-63 cells were seeded in 24-well tissue culture plates (Corning, NY, USA) at a density of 1 × 10^4^ cells/well. After 1 day of culture, the culture medium was replaced with* DBT* extract. After culturing for 2 days, the proliferation and differentiation of osteoblasts were evaluated by 3-(4,5-dimethylthiazol-2-yl)-2,5-diphenyltetrazolium bromide (MTT, Sigma-Aldrich, St. Louis, MO, USA) assay and alkaline phosphatase (ALP) activity assay, respectively, as described below [5].

Murine monocyte/macrophage RAW 264.7 cells (BCRC number 60001) were obtained from FIRDI. 2 × 10^3^ cells/well RAW 264.7 cells were cultured in Dulbecco's Modified Eagle's medium (DMEM, Gibco) supplemented with 5% FBS and 1% penicillin/streptomycin in a humidified 5% CO_2_ incubator at 37°C. After 1 day of culture, osteoclast differentiation from RAW 264.7 cells was induced with 50 ng/mL RANKL (Alexis Biochemicals, Lausen, Switzerland) in *α*-minimal essential medium (*α*-MEM, Gibco) with 2% FBS for 6 days. The cells were also treated with various concentrations of* DBT* added at different periods.* DBT* was added to the cells from the start of the culture to day 6 (group 1) or from day 7 to day 8 (group 2) [6]. The culture medium was refreshed every 2 days. The proliferation and differentiation of osteoclasts were examined by MTT assay and tartrate-resistant acid phosphatase (TRAP) activity assay, respectively.

### 2.3. MTT Assay for Cell Viability

The proliferation of bone cells was evaluated by MTT assay. After culture, cells were incubated with 10 *μ*L MTT solution (5 mg/mL) and 100 *μ*L culture medium for 4 h at 37°C to form insoluble formazan crystals. The formazan crystals were then dissolved by adding 100 *μ*L of acid isopropyl alcohol (0.04 M HCl in isopropyl alcohol). The concentration of formazan crystals formed in the viable cells was estimated by measuring the absorbance at 570 nm on a multiwell scanning spectrophotometer (MRX Microplate Reader, Dynatech Laboratories Inc., Chantilly, USA) [14]. All experiments were performed in triplicate.

### 2.4. Analysis of ALP for Osteoblast Differentiation

The differentiation of osteoblasts was determined by ALP activity assay as described elsewhere [15]. Briefly, the cells were treated with 20 *μ*L/well 0.1% Triton X-100 (Sigma) for 5 min at room temperature for cell lysis. 100 *μ*L/well of the ALP assay kit (procedure number DG1245-K, Sigma-Aldrich) was then added to produce* p*-nitrophenol from the hydrolysis of* p*-nitrophenyl phosphate. The ALP activity of cell lysates was determined by measurement of absorbance at 405 nm caused by* p*-nitrophenol using a MRX Microplate Reader. Each experimental condition was repeated three times.

### 2.5. Quantifying Bone Nodules via von Kossa Stain

The formation of the mineralized nodules was confirmed using the von Kossa stain [16]. Briefly, 5 × 10^4^ cells/well cultured MG-63 cells were added to the culture medium supplemented with 50 *μ*g/mL L-ascorbic acid (Sigma), 10 mM *β*-glycerol phosphate (Sigma), and 10 nM dexamethasone (Sigma). The medium was mixed with various* DBT* concentrations. The medium was changed every 3 days. After 14 days of culture, cultures were fixed in 2% glutaraldehyde for 20 min. The fixed plates were stained with 5% silver nitrate (Union Chemical Works, Ltd., Hsinchu, Taiwan) for 30 min in darkness, exposed to ultraviolet light for 1 h, and then treated with 5% sodium thiosulfate (Union Chemical Works, Ltd.) for 2 min. After washing, the cells are counterstained with 0.1% nuclear fast red (Sigma) dissolved in 5% aluminum sulfate (JT Baker, Phillipsburg, NJ, USA) for 5 min. The number of mineralized bone nodules was counted under an inverted optical microscope (Axiovert 25, Carl Zeiss, Inc., Goettingen, Germany).

### 2.6. Western Blot Analysis

4 × 10^5^ cells/well cultured MG-63 cells were seeded to osteogenic medium with various concentrations of* DBT* in a 6-well culture plate. The medium was replaced every 3 days. After culturing for 7 days, adherent cells were washed and immersed in ice-cold lysis buffer containing 50 mM Tris (pH 7.5), 1 mM EDTA (pH 7.5), 500 mM NaCl, 10% glycerol, 1 mM *β*-mercaptoethanol, 1% IGEPAL-630/Nonidet P-40, and proteinase inhibitor cocktail (Roche, Basel, Switzerland) [17]. After 30 min of immersion, the cellular lysates were centrifuged at 12000 g for 20 min. The concentration of protein was measured using a BCA protein assay kit (Pierce, Rockford, IL, USA). Equal amounts of protein were separated by 12% sodium dodecyl sulfate polyacrylamide gel electrophoresis (SDS-PAGE) and transferred to nitrocellulose membranes. Nonspecific protein binding was blocked with 5% nonfat milk in PBS for 1 h and then incubated with primary antibodies at 1 : 1000 dilutions for 2 days. The membranes were washed to remove unbound antibodies and then incubated with the secondary antibody diluted at 1 :  1000 for 90 min. The blots were visualized by chemiluminescence using the ECL kit (Pierce) with X-ray film (Konica Minolta, Japan).

### 2.7. Cell Migration in a Wound-Healing Assay

Wound-healing assay was employed to detect the migration effect of* DBT* on osteoblasts. Briefly, transparent adhesive tape with 0.1 cm of wide (3M, St. Paul, MN, USA) was applied on the 12-well tissue culture plates and exposed to UV light for 1 h. After washing three times with PBS, 3 × 10^5^ cells/well of cultured MG-63 cells were seeded in the culture plate. After 1 day of culture, the tape was removed to produce 1 mm gap (wound). After rinsing three times with *α*-MEM, the cells were cultured with various concentrations of* DBT* for 2 days. The cell layers were rinsed with PBS, fixed in 2% glutaraldehyde, and stained with Liu's stain solution (Chin Pao Co., Ltd., Taipei, Taiwan). The degree of cells migration was examined using an inverted optical microscope.

### 2.8. TRAP Analysis and TRAP Stain for Osteoclast Differentiation

Several studies have demonstrated that the formation of mature osteoclasts requires 6 days [18, 19]. After 6 days (group 1) or 8 days (group 2) of culture, TRAP activity was assessed by measuring the amount of TRAP released from osteoclasts using a TRAP assay kit (procedure number 435, Sigma). Briefly, 30 *μ*L culture media was mixed with 100 *μ*L TRAP reagent. Absorbance at 405 nm corresponded to the formation of* p*-nitrophenol that was observed using a MRX Microplate Reader. Each experimental condition was repeated three times.

Osteoclasts in the culture were also observed by using TRAP stain [20]. Briefly, cells were fixed using citrate/acetone fixative solution for 30 s, followed by rinsing twice with deionized water. The cells were then incubated in the dark using a 300 *μ*L of TRAP stain reagent (procedure number 387A, Sigma) at 37°C for 1 h. After washing twice, cells were counterstained by hematoxylin solution and observed using an inverted optical microscope.

### 2.9. Statistical Analysis

All quantitative data were expressed as means ± standard deviations. Statistical analysis was done using one-way analysis of variance followed by* post hoc* Fisher's LSD test for multiple comparisons. *P* values lower than 0.05 were considered of statistical significance.

## 3. Results

### 3.1. Effects of DBT Concentration on Osteoblast

**Figure 1:**
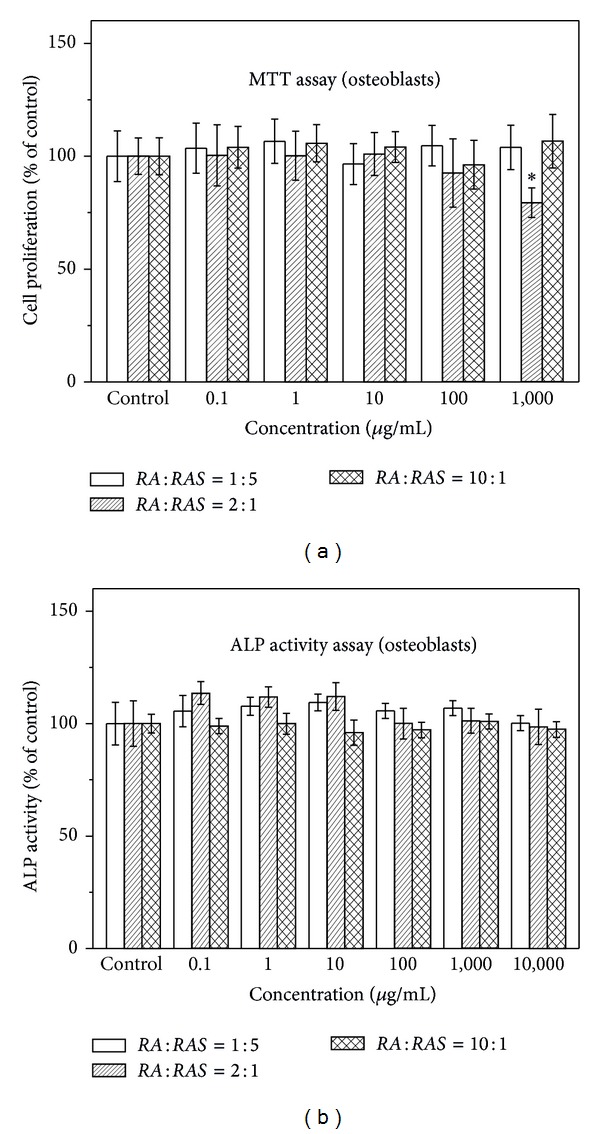
Effect of* DBT* extract prepared at various ratios of* Radix Astragali* (*RA*) and* Radix Angelicae Sinensis* (*RAS*) (1 : 5, 2 : 1, and 10 : 1) on osteoblast proliferation and differentiation by (a) MTT assay and (b) ALP activity assay, respectively. Results are expressed as percentage of control (*P < 0.05 versus control).

**Figure 2:**
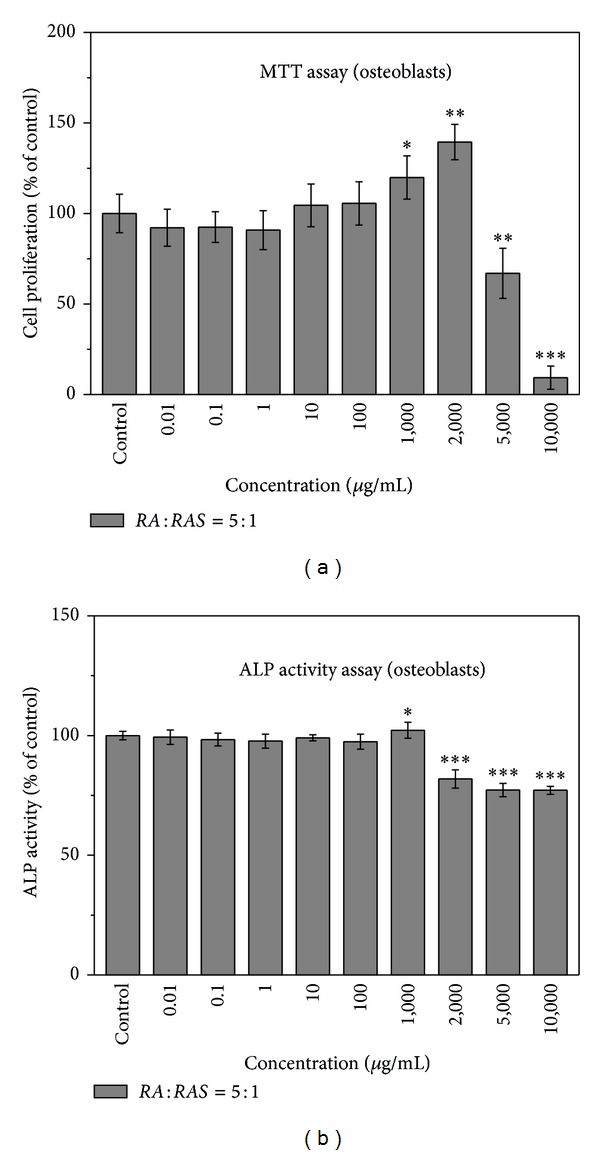
Effect of* DBT* extract prepared from* Radix Astragali* and* Radix Angelicae Sinensis *at a ratio of 5 : 1 on osteoblast proliferation and differentiation by (a) MTT assay and (b) ALP activity assay, respectively. Results are expressed as percentage of control (*P < 0.05, **P < 0.01, and ***P < 0.001 versus control).

The proliferation of osteoblasts induced by different ratios of* RA* to* RAS *in* DBT *and various concentrations of* DBT* was quantified by MTT assay.* DBT* extracted from* RA* and* RAS* in ratios of 1 : 5, 2 : 1, and 10 : 1 did not significantly influence the proliferation of osteoblasts at all concentrations, 0.1–1,000 *μ*g/mL, except that 1,000 *μ*g/mL of* DBT* extracted from* RA* and* RAS* at a ratio of 2 : 1 significantly decreased the number of osteoblasts (Figure 1(a)). However,* DBT *prepared from* RA* and* RAS* at a ratio of 5 : 1 significantly affected the proliferation of osteoblasts in a dose-dependent manner (Figure 2(a)).* DBT* significantly increased the number of osteoblastic cells at the concentrations between 1,000 and 2,000 *μ*g/mL (*P* < 0.05). However,* DBT* significantly inhibited osteoblast growth when the concentration of* DBT* was >5,000 *μ*g/mL (*P* < 0.01).

ALP localized on the cell membrane of osteogenic cells was assessed by ALP activity assay.  Figure 1(b) shows that* DBT* prepared from* RA* and* RAS* in ratios of 1 : 5, 2 : 1, and 10 : 1 had no statistical difference in the ALP activity. However, various concentrations of* DBT *prepared from* RA* and* RAS* at a ratio of 5 : 1 had different effects on the ALP activity of MG-63 cells (Figure 2(b)). Compared with the control, 1,000 *μ*g/mL of* DBT* significantly increased osteoblastic cell differentiation (*P* < 0.05). However, the ALP activity significantly reduced when the concentration of* DBT* was > 2,000 *μ*g/mL (*P* < 0.001). Therefore,* DBT* prepared from* RA* and* RAS* in ratios of 1 : 5, 2 : 1, and 10 : 1 was not evaluated in the following study. Moreover, concentrations higher than 1,000 *μ*g/mL for* DBT* prepared from* RA* and* RAS* at a ratio of 5 : 1 were also not investigated in the following study except Western blot analysis.

**Figure 3:**
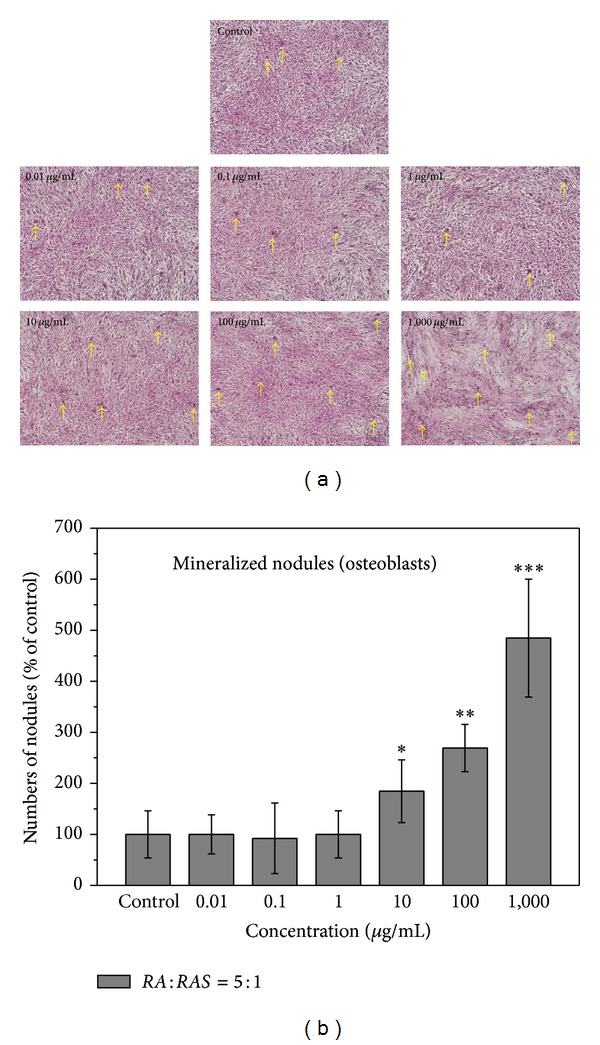
Effect of* DBT *extract prepared from* Radix Astragali* and* Radix Angelicae Sinensis* at a ratio of 5 : 1 on (a) matrix calcium deposition and (b) numbers of total calcified nodules formed in the osteoblast cultures at various concentrations of* DBT*, as determined by von Kossa stain. Results are expressed as percentage of control (*P < 0.05, **P < 0.01, and ***P < 0.001 versus control). Arrows demonstrate deposition of mineralized matrix.

Figure 3 demonstrates the effect of various concentrations of* DBT *prepared from* RA* and* RAS* at a ratio of 5 : 1 on calcium deposition stained with von Kossa stain. 1,000 *μ*g/mL of* DBT* had higher percentage of areas of calcium nodules to total area than all of the other concentrations, 0–100 *μ*g/mL (Figure 3(a)). Moreover, compared with control,* DBT* significantly increased the number of total nodules formed when the concentration of* DBT* was > 10 *μ*g/mL (*P* < 0.05). In particular, 1,000 *μ*g/mL of* DBT* significantly raised the number of total calcified nodules by 380% (Figure 3(b)).

**Figure 4:**
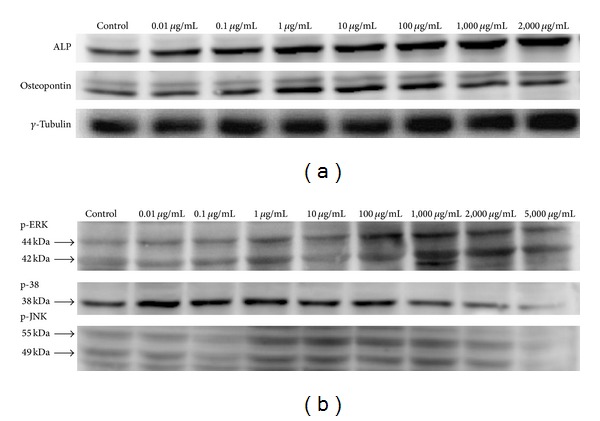
Effect of* DBT* extract prepared from* Radix Astragali* and* Radix Angelicae Sinensis* at a ratio of 5 : 1 on protein expression of (a) alkaline phosphatase, osteopontin, and *γ*-tubulin and (b) p-ERK, p-38, and p-JNK by Western blot analysis.

To determine the effect of* DBT* prepared from* RA* and* RAS* at a ratio of 5 : 1 on osteoblast differentiation, MG-63 cells were treated with various concentrations of* DBT* (0.01–2,000 *μ*g/mL) for 7 days. The expression levels of osteogenic-related proteins, ALP and osteopontin, were then evaluated by Western blot analysis.  Figure 4(a) displays that all ALP, osteopontin, and *γ*-tubulin expression levels on* DBT*-treated osteoblasts were higher than those of the control group. However, the ALP activity assay showed that 2,000 *μ*g/mL of* DBT* inhibited the differentiation of osteoblasts (Figure 2(b)). The difference in the results might be due to different culture periods (2 days versus 7 days) and media compositions used before the ALP activity assay and Western blot analysis were performed.

The mitogen-activated protein kinases (MARKs) regulate cell proliferation, differentiation, motility, and survival in coordination with each other [21]. This study also observed the proliferative effect of* DBT* prepared from* RA* and* RAS* at a ratio of 5 : 1 on the regenerative ability of MG-63 cells cultured with various concentrations of* DBT* (0.01–5,000 *μ*g/mL) for 12 h.  Figure 4(b) reveals that* DBT* had a dose-dependent effect on the expression of MARKs such as p-ERK (about 42 and 44 kDa) and p-JNK (about 49 and 55 kDa). 1,000 *μ*g/mL of* DBT* induced the highest p-ERK expression and higher p-JNK levels. No effects occurred at lower doses, while some declined at higher concentrations. Moreover, the decrease in p-38 phosphorylation was found as p-ERK and p-JNK activity increased. We believe that* DBT* can activate the phosphorylation of p-ERK and p-JNK signal pathway to stimulate the proliferation and differentiation of human osteosarcoma cell line MG-63.

**Figure 5:**
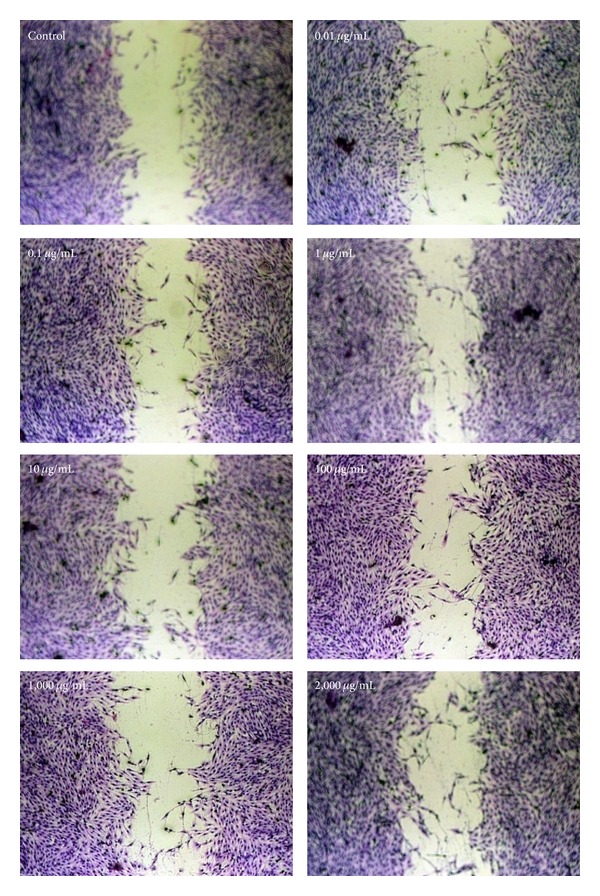
Effect of* DBT* extract prepared from* Radix Astragali* and* Radix Angelicae Sinensis* at a ratio of 5 : 1 on the migratory ability of osteoblasts, as determined by wound-healing assay.

The ability of osteoblastic cell to migrate along the growth direction was examined by an* in vitro* wound-healing experiment. Compared with the control, 0.01–2,000 *μ*g/mL of* DBT* prepared from* RA* and* RAS* at a ratio of 5 : 1 markedly enhanced the mobility of MG-63 cells (Figure 5). Moreover,* DBT* induced osteoblastic cell proliferation. These results indicate that* DBT* could enhance bone cell regeneration.

### 3.2. Effects of DBT Concentration on Osteoclast

**Figure 6:**
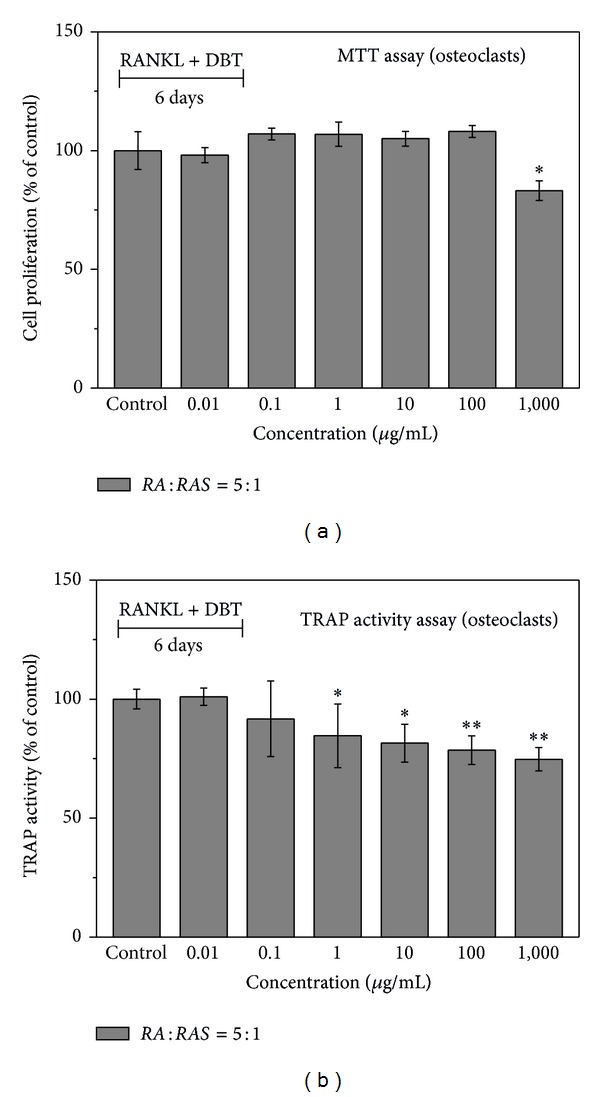
Effect of* DBT* extract prepared from* Radix Astragali* and* Radix Angelicae Sinensis* at a ratio of 5 : 1 on osteoclast proliferation and differentiation by (a) MTT assay and (b) TRAP activity assay, respectively, after various concentrations of* DBT* extract were added for 6 days (proliferative and differentiation phases). Results are expressed as percentage of control (*P < 0.05 and **P < 0.01 versus control).

The RAW 264.7 cells were used to evaluate the osteoclastogenic effect of* DBT *prepared from* RA* and* RAS* at a ratio of 5 : 1. In group 1 (proliferative and differentiation phases), various concentrations of* DBT* and 50 ng/mL of soluble RANKL were applied onto the cultured RAW 264.7 cells for 6 days to induce the differentiation of monocytes/macrophages into osteoclasts.  Figure 6(a) displays how various doses (0.01–1,000 *μ*g/mL) affect the proliferation of osteoclasts measured by MTT assay. Consequently, no statistically significant difference from the control group was observed at the lower concentration of 0.01–100 *μ*g/mL. Conversely,* DBT* significantly lowered the proliferation of osteoclasts at 1,000 *μ*g/mL (*P* < 0.05). Moreover, the TRAP activity of osteoclasts decreased when adding* DBT* at concentrations of 1–1,000 *μ*g/mL (*P* < 0.05) (Figure 6(b)). When* DBT* inhibited TRAP activity, the number of osteoclasts was lower than the control group (Figure 8(a)).

**Figure 7:**
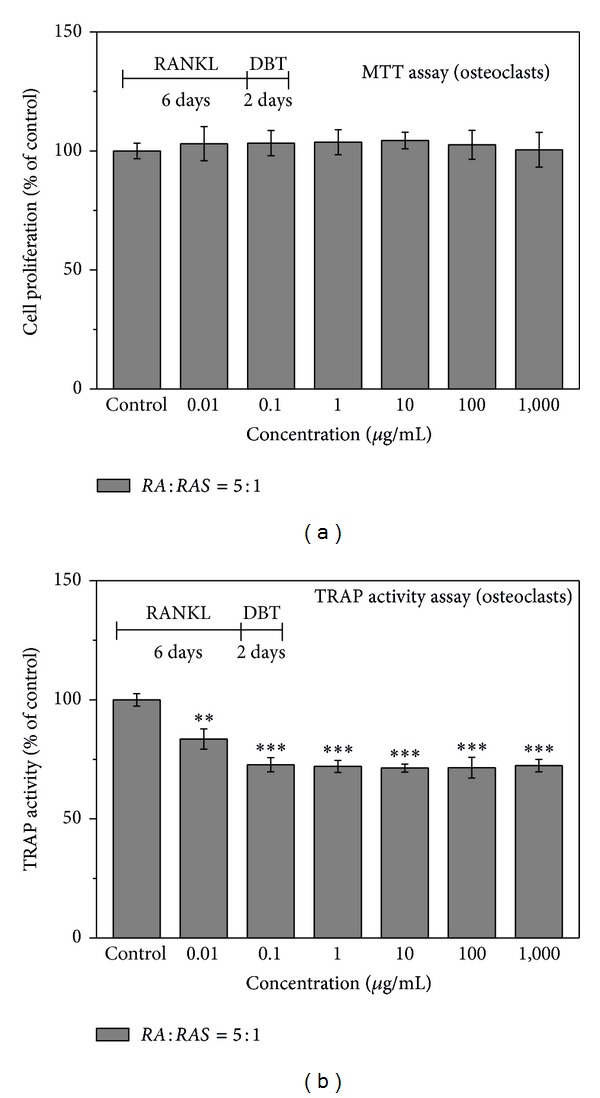
Effect of* DBT* extract prepared from* Radix Astragali* and* Radix Angelicae Sinensis* at a ratio of 5 : 1 on osteoclast proliferation and differentiation by (a) MTT assay and (b) TRAP activity assay, respectively, after various concentrations of* DBT* extract were added for 2 days at day 7 to 8 (mature phase). Results are expressed as percentage of control (**P < 0.01 and ***P < 0.001 versus control).

**Figure 8:**
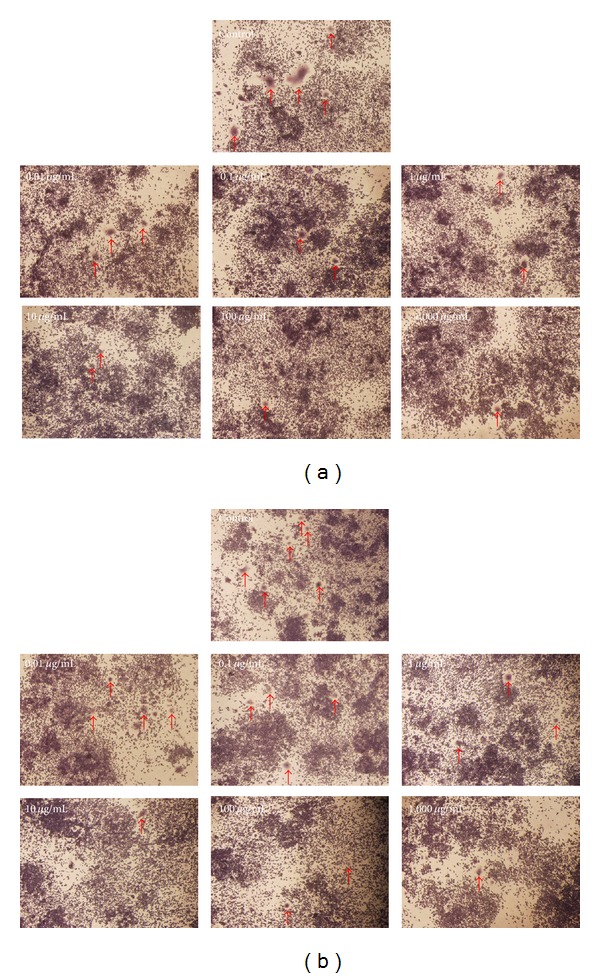
TRAP staining of osteoclasts treated with different concentrations of* DBT *extract (a) for 6 days and (b) for 2 days at day 7 to 8. Arrows demonstrate osteoclasts.

For a closer examination (group 2, mature phase), after RAW 264.7 cells were treated with 50 ng/mL RANKL for 6 days,* DBT* was then added to the mature osteoclasts from day 7 to day 8 (for 2 days).  Figure 7(a) clarifies that* DBT* did not affect the proliferation of mature osteoclasts. TRAP activity assay revealed that* DBT* at concentrations of 0.01–1,000 *μ*g/mL produced significant decreases in TRAP activity (Figure 7(b)). When* DBT* inhibited TRAP activity, the number of osteoclasts was lower than the control group (Figure 8(b)). These results suggest that* DBT* can inhibit the RANKL-induced osteoclast differentiation of RAW 264.7 cells.

## 4. Discussion

Several studies have documented the feasibility of alleviating bone disorders and liver diseases following treatment with Chinese herbal decoction* DBT* [8–11, 13]. Specific biological advantages, which can be achieved from Chinese medicine, must include faster and more uniform bone ingrowth [3]. As is well known, osteoblasts and osteoclasts in the fracture site are actively engaged in the synthesis and secretion of collagen [22]. To repair skeletal defects, osteoblasts should populate the defects by proliferation of the transplanted cells and migration of cells into the defect from the surrounding tissue; the construct is ultimately filled by the osteoblasts and healing of large osseous defects [23]. Our previous study developed and evaluated tricalcium phosphate, gelatin, and Chinese medicine as a new bone substitute [19]. During bone repair, bone remodeling involves bone resorption by osteoclasts, which is followed by bone formation by osteoblasts. This study investigates how* DBT* affects bone cell activity.

The results of the biological evaluation indicate that* DBT* prepared from* RA* and* RAS* at a ratio of 5 : 1 had a significant osteotropic effect. Moreover, the optimal concentration of* DBT* prepared from* RA* and* RAS* at a ratio of 5 : 1 was 1,000 *μ*g/mL, which obviously raised the number of osteoblasts, intracellular ALP levels, and nodule numbers, while suppressing osteoclast activity. Additionally, applying* DBT* to osteoblasts triggered the downstream signaling cascades including p-ERK and p-JNK signal pathways. Doing so facilitated the proliferation and differentiation of human osteosarcoma cell line MG-63, thus demonstrating excellent osteoinductive activity. Moreover,* DBT* could inhibit the RANKL-induced osteoclast formation* in vitro*.

Traditional Chinese medicine has been developed empirically based on clinical experience. Importantly, traditional Chinese medicine can be used systemically to accelerate bone formation or diminish bone resorption in order to treat bone diseases. For early stage of healing and resorption remodeling process, individual Chinese medicines (e.g.,* Loranthus parasiticus,* Achyranthes bidentata*, and Drynaria fortunei*) can enhance osteoclast formation by stimulating the proliferation in bone resorption. In the middle and late phases of healing, Chinese medicines such as* Cuscuta chinensis*,* Eucommia ulmoides,* and Dipsacus* asper* can potentially inhibit osteoclast proliferation and promote osteoblastic proliferation and differentiation [6, 19].

## 5. Conclusion

This work demonstrates the biological functions of this decoction in promoting the proliferation, differentiation, and mineralization of osteoblasts* in vitro* as well as inhibiting osteoclast activity. Importantly,* DBT* is highly promising for use in accelerating fracture healing in the middle or late healing periods and treating osteoporosis.
